# Anévrysme de l'artère hépatique révélé par une pancréatite - à propos d'un cas et revue de la literature

**DOI:** 10.11604/pamj.2014.18.324.5108

**Published:** 2014-08-21

**Authors:** Mehdi Soufi, Abdelatif Settaf, Bouziane Mohammed, Tijani Harroudi, Rahal Messrouri, Jalil Mdaghri, Ahmed Taghy, Bouziane Chad

**Affiliations:** 1 Département de Chirurgie, Faculté de Médecine d'Oujda, Université Mohammed premier, Oujda, Maroc; 2 Service de Chirurgie Viscérale B, CHU Avicenne, Rabat, Maroc

**Keywords:** Anévrysme, artère hépatique, chirurgie, embolisation, aneurysm, hepatic artery, surgery, embolization

## Abstract

Les anévrysmes de l'artère hépatique sont rares et pourvoyeurs de complications graves. La pancréatite est reste une mode de révélation inhabituel. À travers une observation d'un anévrysme de l'artère hépatique propre et les auteurs font une mise au point sur les anévrysmes de l'artère hépatique, les auteurs discutent le rôle de la chirurgie et le rétablissement du flux hépatique artériel dans le traitement de ces lésions vasculaires.

## Introduction

Les anévrysmes des artères digestives sont rares [1]. Ils intéressent l'artère hépatique dans environ 20% des cas [2]. Les étiologies sont nombreuses dont la pancréatite est reste une mode de révélation inhabituel des anévrysmes de l'artère hépatique [2]. Cette affection est de plus en plus découverte par les progrès de l'imagerie [1]. Le risque de rupture élevé de ces anévrysmes impose un traitement rapide et efficace. Diverses options thérapeutiques sont disponibles pour traitement de cette pathologie. Le maintien de la perfusion distale du foie est doit être pris en considération pour toute décision thérapeutique [1, 2]. Nous rapportons un cas d'anévrysmes de l'artère hépatique propres révélé par une pancréatite et traité chirurgicalement par deux rétablissements de la perfusion artérielle. A travers cette observation, nous discuterons les modalités cliniques et diagnostiques et la place de la chirurgie dans le traitement de cette affection.

## Patient et observation

**Figure 1:**
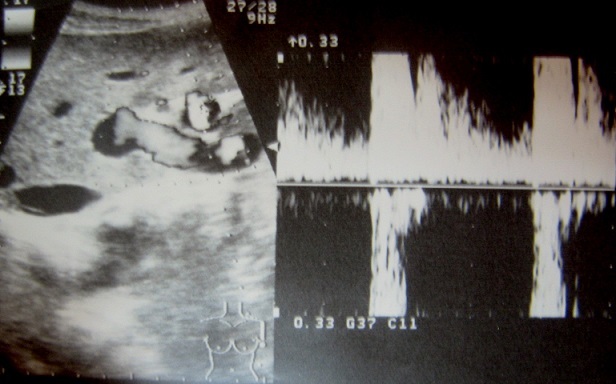
Écho-endoscopie: artère hépatique propre siège d'un anévrysme

**Figure 2:**
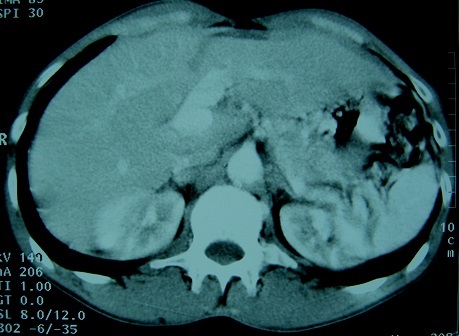
Angioscanner: anévrysme de 2,5 cm de diamètre partiellement thrombosé

**Figure 3:**
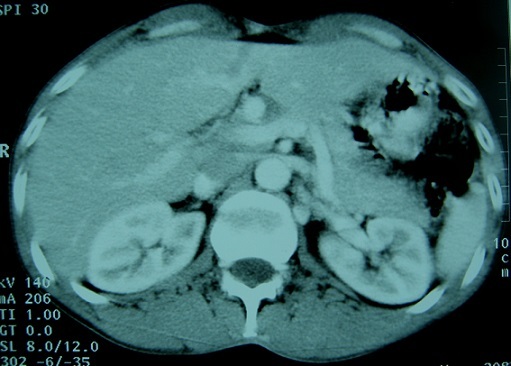
Angiographie: anévrysme de l'artère hépatique propre qui prend l'origine de l'artère gastroduodénale et la branche droite et gauche d e l'artère hépatique

**Figure 4:**
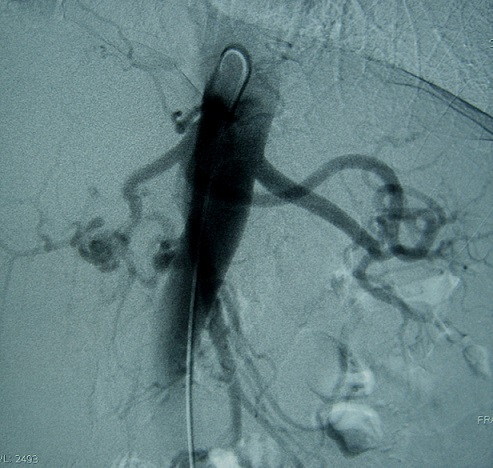
Aspect peropératoire après dissection du sac anévrysmale

**Figure 5:**
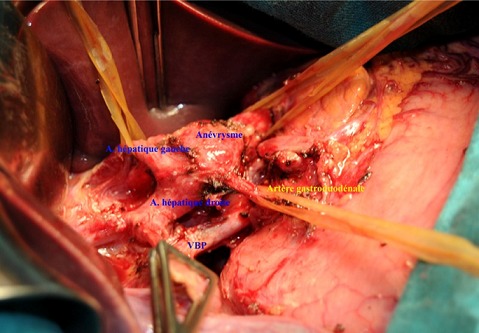
Confection de deux anastomoses entre l'artère hépatique commune et la branche gauche de l'artère hépatique et entre l'artère gastroduodénale et la branche droite de l'artère hépatique

**Figure 6:**
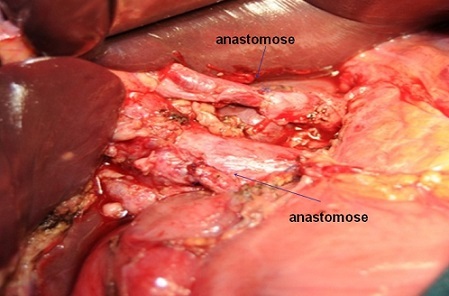
Pièce de résection confection de deux anastomoses entre l'artère hépatique commune et la branche gauche de l'artère hépatique et entre l'artère gastroduodénale et la branche droite de l'artère hépatique

**Figure 7:**
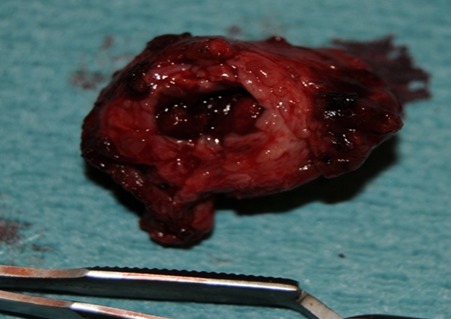
Sacs anévrysmale: pièce de résection

Les auteurs déclarent avoir reçu le consentement écrit du patient pour reporter ce cas. Un homme âgé de 44 ans, tabagique chronique à raison de 15 PA, se plaignait depuis trois semaines des douleurs épigastriques et de l'hypochondre droit. Deux jours avant l'hospitalisation, la symptomatologie s’était accentuée avec l'installation de vomissements bilieux. L'examen clinique était normal. Le bilan biologique montrait une hémoglobine à 11,9 g/dL, une hyperleucocytose à 13 000 GB/mm^3^, une lipasémie à 3,5 fois la normale (196.3 U/L) et un ionogramme sanguin normal. Une fibroscopie oesogastroduodénale était sans particularité. Une échographie révélait l'existence d'une vésicule biliaire lithiasique avec présence d'une image kystique de la tête du pancréas. Une écho-endoscopie montrait la présence d'un anévrysme de l'artère hépatique propre de 2,5 cm de diamètre (Figure 1). L'angioscanner confirmaient qu'il s'agissait d'un anévrysme de l'artère hépatique partiellement thrombosé (Figure 2). L'artériographie coeliomésentérique montrait que l'anévrysme était localisé au niveau de l'artère hépatique propre s’étendant à l'artère gastroduodénale et à la bifurcation de l'artère hépatique (Figure 3). Par une voie d'abord bisouscostale, la dissection du pédicule hépatique trouvait un anévrysme de l'artère hépatique propre de 2,5 cm qui prenait l'origine de l'artère gastroduodénale et de la branche hépatique droite et gauche (Figure 4). Le geste opératoire a consisté en une cholécystotomie avec résection du sac anévrysmale, suivi deux anastomoses artérielles (Figure 5, Figure 6, Figure 7). Une entre l'artère hépatique commune et la branche gauche de l'artère hépatique, et l'autre entre l'artère gastroduodénale et la branche droite de l'artère hépatique, étant donné qu'il existait un reflux sanguin issu de l'artère gastroduodénale. Les suites opératoires étaient simples. Le contrôle par echodoopler à un mois montrait la perméabilité des anastomoses avec un foie normal.

## Discussion

Décrite la première fois par Wilson en 1819 [1], les anévrysmes de l'artère hépatique sont rares mais graves. Il touche le plus souvent l'homme de la quatrième décennie, avec un sex-ratio de 2/1 [2]. Considéré depuis longtemps comme, l'anévrysme le plus fréquent après celui de l'artère splénique, il existe récemment une recrudescence de cette affection tendant à devenir l'anévrysme le plus fréquent des artères digestives [2]. Ceci est probablement du au progrès de l'imagerie médicale et au développement des centres hépatobiliaires [3]. Environ 80% des anévrysmes sont extra hépatiques. Ils peuvent être unique ou multiples [3]. Ils touchent essentiellement l'artère hépatique commune dans les deux tiers des cas et l'artère hépatique droite dans 28% des cas [1, 3]. La localisation gauche ou bilatérale est beaucoup plus rare [2, 3]. Les causes sont nombreuses, l'athérosclérose représente l’étiologie la plus fréquente (33%). Les autres étiologies sont l'altération de la média, la dysplasie fibromusculaire, et plus rarement le lupus érythémateux disséminé, le syndrome d'Ehlers-Danlos, la maladie de Buerger, la maladie de Kawasaki et la sarcoïdose [4]. Avec l'utilisation fréquente des antibiotiques, les anévrysmes d'origine infectieuse et mycotique ne représentent plus aujourd'hui que 10% des anévrysmes de l'artère hépatique [1, 4]. L'inflammation périartérielle suite à une pancréatite aiguë peut être incriminée [3, 4].

Il existe trois types d'anévrysmes: les anévrysmes vrais, disséquant et les faux anévrysmes [5]. Cliniquement, la symptomatologie est variable. 60% des anévrysmes sont asymptomatiques. Le mode de révélation le plus commun est la douleur épigastrique. Chez notre patient, la clinque peut être expliquée par l'anévrysme, la lithiase et la pancréatite. D'autres signes peuvent être révélatrice de l'anévrysme, notamment l'hémorragie digestive, l'ictère. L’étiopathogénie est encore mal élucidé [3, 5]. Le risque de rupture atteint 80% dans la littérature avec une mortalité allant de 30% à 40% [3–5]. Il n'existe aucune corrélation entre la taille de l'anévrysme qui peut être géant et le risque de rupture Cette dernière peut se faire dans le péritoine, les voies biliaires, le pancréas, le tube digestif, voire dans la veine porte [5, 6]. Le diagnostic repose sur l'imagerie. Notre observation présente une autre particularité, l’échographie avait évoqué une lésion kystique du pancréas, ce qui a poussé a réalisé une écho-endoscopie qui a confirmé le diagnostic. Une seule étude sur 4 cas a rapporté les descriptions de cet examen dans les anévrysmes [1]. Cette entité demeure importante car à défaut, les lésions peuvent être prises pour des kystes pancréatiques et dont la biopsie à but diagnostiques peut être fatale [2, 7]. Les Doppler pulsé et couleur ont un but diagnostic, en montrant une image hypoéchogène pulsatile, et thérapeutique en guidant une éventuelle embolisation percutanée. Ils permettent aussi de contrôler l'efficacité du traitement et l’évolution de l'anévrysme [8]. La tomodensitométrie et l'angioscanner permettent de faire un bilan lésionnel précis en déterminant les dimensions de l'anévrysme, et en distinguant entre la vraie lumière et le thrombus pariétal [3, 8]. L'exploration vasculaire de choix repose sur l'artériographie qui peut mettre en évidence les vaisseaux nourriciers, les fistules ou d'autres localisations de l'anévrysme. Elle permet ainsi de préciser la décision thérapeutique au dépend de la localisation exacte de l'anévrysme et de la nécessité ou non d'un rétablissement de la vascularisation [9]. En l'absence de l'urgence, la réalisation d'une angio-IRM, technique non invasive, dispense actuellement de la réalisation de l'artériographie. Elle apporte les mêmes données morphologiques et fonctionnelles de l'artériographie en étudiant en plus, par les séquences de cholongio-IRM, le temps biliaire afin de rechercher des complications liées à l'anévrysme [8, 9].

Grâce au progrès de la radiologie interventionnelle et de la chirurgie, le traitement de l'anévrysme est actuellement codifié. Les indications dépendent de la taille, du type, de la localisation de l'anévrysme, de l’état général du malade [9, 10]. Le traitement de choix des anévrysmes est la chirurgie [10]. L'embolisation reste une bonne alternative pour les anévrysmes intra hépatiques, et pour les patients ayant un haut risque chirurgical [11]. Le traitement chirurgical est indiqué si l'anévrysme est symptomatique ou si le diamètre excède 2 centimètres [10]. Vu le risque de rupture tout les anévrysmes non athéroscléreux même asymptomatique doivent être traités [10]. Notre patient avait un anévrysme extrahépatique, dans ces cas, différentes techniques sont rapportées. La ligature, la résection, la greffe veineuse, la greffe prothétique, la résection hépatique [12, 13]. Nous avons réalisé une résection du sac anévrysmale avec confection de deux anastomoses pour rétablissement du flux artériel pour plusieurs raisons: L'anévrysme siégeait en amont et en aval de l'artère gastroduodénale, il s’étendait aux deux branches hépatiques: droite et gauche. Aucune artère de suppléance pour la vascularisation hépatique n'a été retrouvée. Chez les patients inopérables, l'embolisation du sac anévrysmal constitue une bonne alternative un taux de succès remarquable, toutefois, cette technique présente un risque de reperméabilisation avec accroissement de l'anévrysme responsable de complications [11]. Nous pensons que la résection du sac, pour analyse histologique, et le rétablissement de la continuité du flux artériel est le traitement idéal. Nous avons confectionné deux anastomoses termino-terminales vu que les bouts artériels après dissection pouvaient être suturés sans tension. A défaut la mise en place d'un greffon ou d'une prothèse, en dehors de l'infection, avec réimplantation de l'artère gastroduodénale constitue une bonne alternative [10, 12, 13]. Certains auteurs suggèrent, comme option thérapeutique, la ligature artérielle même en aval de l'artère gastroduodénale [14], ils pensent que la restauration du flux n'est pas nécessaire car il existe des collatéraux à destinée hépatique chez 45% de la population [13, 14]. Dans notre cas, aucune artère collatérale n’était visualisé. Vu le risque d'ischémie hépatique fréquemment rapporté après ligature, nous n'utilisons jamais cette méthode.

## Conclusion

Les anévrysmes de l'artère hépatique sont rares et pourvoyeurs de complications graves. La crise de pancréatite aigue est un mode de révélation non commun. Le diagnostic repose sur l'imagerie. La chirurgie est le traitement choix. Chaque fois que possible, le rétablissement du flux artériel hépatique est primordial. En raison la grande variabilité anatomique des artères hépatique. L'attitude thérapeutique doit être discutée au cas par cas. Quand l'anévrysme s’étant à la bifurcation artérielle de l'artère hépatique propre et au tronc de l'artère gastroduodénale, la confection de deux anastomoses entre l'artère hépatique commune et la branche gauche de l'artère hépatique et entre l'artère gastroduodénale et la branche droite de l'artère hépatique représente le meilleur traitement.
